# 

**Published:** 1896-10

**Authors:** 


### CONTENTS.

PROCEEDINGS.

Proceedings of the meeting of the Michigan State Dental Association .......... 469
Fifty-third annual meeting of the Mississippi Valley Association of Dental Surgeons .......... 501
Monthly Digest .......... 487

COMMUNICATIONS.

Report of Committee on Dental Science and Literature .......... 505
Introductory to Discussion on Dental Nomenclature .......... 507

SELECTIONS.

To Keep Metal from Rusting .......... 500
Fooled by his Imagination .......... 504
Physicians Should do less Work .......... 508
An Anomalous Condition .......... 508
What is Man's Natural Food? .......... 509
Alcohol in the Treatment of Disease .......... 511
Milk from Sewage Farms .......... 512
Surgical Use of Cocaine .......... 513
Growths in the Nasal Passages Due to Colds .......... 514
The British Medical Association .......... 514
Cold Burns .......... 514
A Needed Reform .......... 515
Medicine as a Moral Agent .......... 515
Moving toward the Light .......... 516
Fatigue .......... 516
Meeting of the British Medical Association .......... 516

EDITORIAL.

Educational—College Work .......... 517
Bibliograpical .......... 518
Another Dental College .......... 519
A Mistake .......... 520

